# Real-time three-dimensional transthoracic echocardiographic segmental volume analysis: a quantitative and objective tool for assessing regional left ventricle wall motion in patients with ischemic heart disease

**DOI:** 10.1186/s44348-024-00040-3

**Published:** 2024-12-23

**Authors:** Jin-Hwan Kwak, Kang-Un Choi, Jong-Il Park, Jong-Ho Nam, Chan-Hee Lee, Ung Kim, Jong-Seon Park, Jang-Won Son

**Affiliations:** Division of Cardiology, Department of Internal Medicine, Yeungnam University Medical Center, Daegu, Republic of Korea

**Keywords:** Echocardiography, Three-dimensional, Regional left ventricle wall motion, Segmental volume, Myocardial Ischemia, Imaging, Left ventricular function

## Abstract

**Background:**

Evaluation of regional left ventricle function using two-dimensional echocardiography (2DE) in patients with ischemic heart disease has limitations due to its low objectivity and qualitative nature. In addition, 2DE is limited because multiple acoustic windows are used to obtain the image, whereas three-dimensional echocardiography (3DE) uses a single window. This study aims to demonstrate the clinical utility of 3DE segmental volume analysis for evaluating regional wall motion abnormality (RWMA).

**Methods:**

This retrospective study included 33 patients with ischemic heart disease and single-vessel territory RWMA confirmed on coronary angiography. RWMA was visually assessed using 2DE, generating 17-segment bull's-eye polar maps, and 3DE. In the 3DE study, two independent observers analyzed segmental volumes and segmental volume ejection fractions (SVEFs) using QLAB 3D quantification software. The optimal SVEF cutoff value differentiating normal from abnormal was determined using receiver operating curve analysis. The accuracy of 3DE in predicting culprit coronary arteries was compared with that of 2DE using Cohen κ coefficients, which also were used for interobserver and intraobserver variability assessments.

**Results:**

Mean 3DE SVEFs were significantly lower in segments showing RWMA on 2DE. The optimal SVEF cutoff value was 44%, with sensitivity of 75.0% and specificity of 73.9% (area under the curve, 0.801; 95% CI, 0.763–0.838; *P* < 0.001). The reliability of 3DE-derived bull's-eye predictions of culprit coronary arteries was 81.8% (κ = 0.672; 95% CI, 0.555–0.789; *P* < 0.001). Interobserver and intraobserver variabilities were 97.0% (κ = 0.947; 95% CI, 0.894–1.00; *P* < 0.001) and 93.9% (κ = 0.897; 95% CI, 0.827–0.967; *P* < 0.001), respectively.

**Conclusions:**

The 3DE segmental volume analysis effectively quantified regional left ventricle function and aligned well with 2DE and coronary angiography findings in predicting culprit coronary arteries. Thus, 3DE segmental volume analysis can serve as a quantitative and objective tool for RWMA assessment in patients with ischemic heart disease.

## Background

Two-dimensional echocardiography (2DE) is the most commonly accessible and indispensable non-invasive imaging modality for assessing regional left ventricle (LV) function in patients with ischemic heart disease (IHD), for whom immediate and accurate diagnosis is crucial. However, 2DE is limited because it is subjective, semiquantitative, and expert-dependent [1, 2]. Another drawback of 2DE is that multiple acoustic windows are required to acquire images.

However, three-dimensional echocardiography (3DE) has the advantages of acquiring the entire LV from a single apical window and enabling rapid LV volume measurement without geometric assumptions [3]. In addition, the measurement method offers the advantage of acquiring images from locations less affected by bone structures or patient positioning, which can potentially lead to more consistent and reliable imaging results.

To assess regional wall motion abnormality (RWMA) using volume, which has an objective and quantitative property, an algorithm that can semiautomatically recognize LV endocardium and compute segmental volumes without geometrical assumptions was devised. Using this algorithm, the mean segmental volume ejection fraction (SVEF) was lower for LV segments having RWMA on 2DE [4]. The goals of this study were as follows: (1) determine a difference in SVEF by 2DE wall motion score; (2) identify the 3DE SVEF cutoff value that best predicted the presence of RWMA; and (3) demonstrate the usefulness of 3DE SVEF in the assessment of the culprit coronary artery in patients with IHD.

## Methods

### Study subjects

We retrospectively reviewed 39 consecutive patients who underwent echocardiography followed by invasive coronary angiogram. If emergency coronary angiogram was required, echocardiography was performed within 24 h. Inclusion criteria were a single-territory RWMA on 2DE and single-vessel obstructive coronary artery disease confirmed on coronary angiography; no valvular stenosis or greater than mild regurgitation; normal sinus rhythm without left bundle branch block; and no previous history of myocardial infarction. After excluding 6 patients with an inadequate image, low 3D image frame rate, or low resolution, 33 patients were enrolled in the current investigation. If RWMAs were discovered on 2DE, 3DE was performed for further analysis.

### Echocardiographic examination

Conventional 2DE examinations were performed according to the most recent American and European echocardiography guidelines [1] using an X5-1 phased-array xMatrix transducer (X5-1 Transducer, Philips Healthcare) and cardiovascular ultrasound system (EPIQ CVx ver. 7.0, Philips Healthcare). As proposed by the American Society of Echocardiography (ASE) and the European Association of Cardiovascular Imaging (EACVI), LVs were divided into 17 segments to assess regional function [1, 5]. The 2D regional LV function was assessed using echocardiography based on observed wall thickness and endocardial motions of the myocardial segments. Each segment was evaluated in multiple views to assess regional function and tagged individually using a 4-point scoring system [1]: 1, normal; 2, hypokinetic (reduced thickening); 3, akinetic (absent or negligible thickening); and 4, dyskinetic (systolic thinning or stretching). The same probe was used to acquire the 3D image.

### 3D regional LV function analysis

Seventeen SVEFs were individually calculated for each patient in two stages. The first was 3D LV segmentation using a QLAB 3D Quantification Advanced software ver. 10.0 (Philips Healthcare) to conduct deformable modeling without geometric assumptions [6]. The second was SVEF calculations. four mitral annular and apical points were placed on the LV, and the location of the mid interventricular septum is designated to produce an output time-volume graph for each of the 17 segments. The segmental volume versus time data were exported as a Microsoft Excel file (Microsoft Corp). For each segment, the largest and smallest volumes were defined as end-diastolic volume (EDV) and end-systolic volume (ESV), respectively. Regional SVEF values were manually calculated using the formula below:

$$\text{SVEF }= (\text{EDV }-\text{ ESV}) /\text{ EDV }\times 100 (\text{\%})$$

Next, SVEF values were calculated for each segment in each patient. For each segment, 2DE-derived wall motion scores (4-point scoring system) were individually matched in succession with 3DE SVEF values.

Analysis of variance (ANOVA) was used to determine whether 3DE SVEF could differentiate between 2DE-derived wall motion score groups.

#### Determining a 3DE SVEF cutoff value

Based on each 3DE SVEF, the optimal cutoff point to best discriminate the 2DE wall motion score was calculated using receiver operating curve (ROC) analysis with IBM SPSS ver. 19 (IBM Corp). Because a cutoff value has not been reported for abnormal 3DE SVEF, two models were developed: model 1 using a universal fixed SVEF value for all 33 patients and model 2 using the ratio between 3DE SVEF and individual's 3DE global ejection fraction (EF) value as a parameter. An ROC curve was created for each model, and the areas under the curve (AUCs) were compared.

#### Comparison of estimated culprit arteries determined using 2DE and 3DE bull's-eye maps

The 3DE bull's-eye polar maps were generated using a two-color scale. Individual LV segments were tagged red if their SVEFs were equal to or greater than the cutoff value and grey if they were lower than the cutoff.

**Fig. 1:**
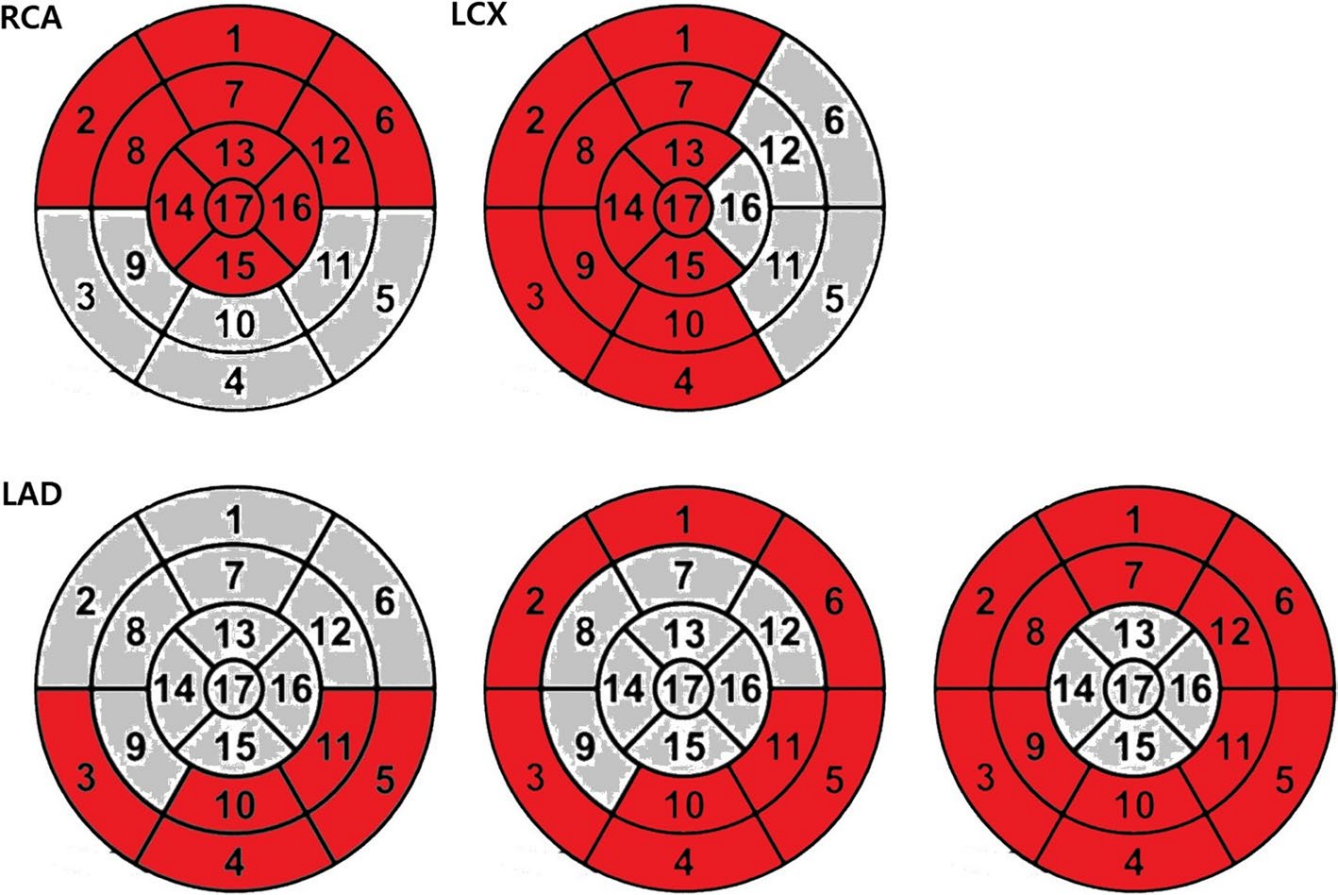
Example of bull's-eye polar map showing the typical coronary artery distribution established by the American Society of Echocardiography and European Association of Cardiovascular Imaging [1]. The segments that could show RWMA were marked in grey, while those without RWMA were marked in red. RCA, right coronary artery; LCX, left circumflex coronary artery; LAD, left anterior descending artery; RWMA, regional wall motion abnormality

**Fig. 2:**
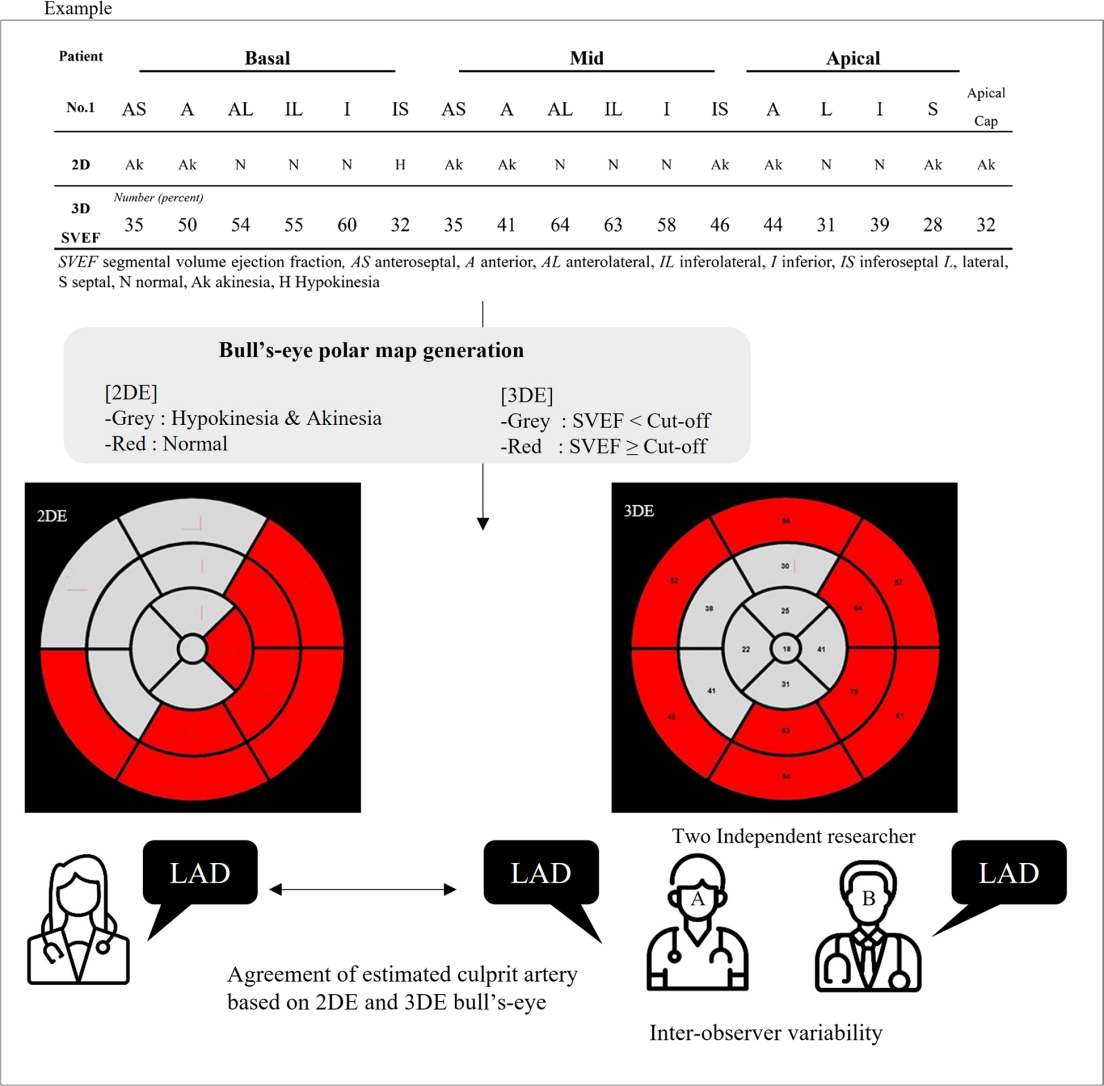
Overview of the comparison process for estimating culprit arteries determined using two-dimensional echocardiography (2DE) and three-dimensional echocardiography (3DE) bull's-eye maps. AS, anteroseptal; A, anterior; AL, anterolateral; IL, inferolateral; I, inferior; IS, inferoseptal; L, lateral; S, septal; Ak, akinesia; N, normal; H, hypokinesia; SVEF, segmental volume ejection fraction; LAD, left anterior descending artery

Culprit arteries were estimated in 2DE and 3DE images by an independent researcher using bull's-eye polar maps and typical coronary artery distributions established by the ASE and EACVI (Fig. 1) [1]. Cohen κ coefficients were used to assess the reliability of bull's-eye–derived culprit coronary artery prediction results based on 3DE compared with 2DE (Fig. 2).

#### Interobserver and intraobserver variability

Interobserver variabilities of processes for creating 3D endocardial shell and SVEF analysis were determined using intraclass correlation coefficients (ICCs). Interobserver and intraobserver variabilities of 3DE-derived bull’s-eye culprit coronary artery prediction were determined using Cohen κ coefficients.

## Results

### Clinical characteristics and echocardiographic parameters

The clinical characteristics and echocardiographic parameters of the 33 study subjects are shown in Table 1: Mean patient age was 64.2 ± 12.1 years, 27 (81.8%) were male, 13 (39.4%) had hypertension, and 10 (30.3%) had diabetes. Mean body mass index was 23.07 ± 3.3 kg/m^2^, left ventricular ejection fraction (LVEF) was 48.5% ± 8.1%, and mean wall motion score index was 1.48 ± 0.30. The mean frame rate for full-volume 3DE datasets was 17 ± 3 Hz.

**Table 1:** Baseline characteristics and echocardiographic parameters for 2DE

Characteristic	Value (*n* = 33)
Male sex	27 (81.8)
Age (yr)	64.2 ± 12.1
Height (cm)	164.3 ± 7.5
Weight (kg)	64.3 ± 11.2
Body mass index (kg/m^2^)	23.7 ± 3.3
Risk factor	
Hypertension	13 (39.4)
Diabetes	10 (30.3)
Congestive heart failure	7 (21.2)
Cerebrovascular accident	1 (3.0)
Systolic blood pressure (mmHg)	119 ± 17
Diastolic blood pressure (mmHg)	76 ± 13
Heart rate (bpm)	72 ± 15
Clinical diagnosis	
STEMI	19 (57.6)
NSTEMI	9 (27.3)
Stable angina	5 (15.2)
Diseased vessel	
Left anterior descending artery	20 (60.6)
Left circumflex artery	5 (15.1)
Right coronary artery	8 (24.2)
Echocardiographic parameter	
LVEF (%)	48.5 ± 8.1
LVESD (mm)	38.0 ± 5.8
LVEDD (mm)	51.4 ± 4.9
LVMI (g/m^2^)	105.1 ± 32.2
LAVI (mm^3^/m^2^)	25.3 ± 11.0
E' velocity (cm/sec)	0.05 ± 0.01
E velocity (m/sec)	0.61 ± 0.14
A velocity (m/sec)	0.73 ± 0.19
Wall motion score index	1.48 ± 0.30

Values are presented as number (%) or mean ± standard deviation. Percentages may not total 100 due to rounding

*2DE* two-dimensional echocardiography, *STEMI* ST-segment elevation myocardial infarction, *NSTEMI* non-ST-segment elevation myocardial infarction, *LVEF* left ventricular ejection fraction, *LVESD* left ventricular end-systolic dimension, *LVEDD* left ventricular end-diastolic dimension, *LVMI* left ventricular mass index, *LAVI* left atrial volume index

### Distribution of regional wall motion scores by segment determined using 2DE

The distribution of regional wall motion scores by segment determined on 2DE is shown in Table 2. Because there was no segment for dyskinesis, the regional wall motion group was separated into three subgroups: normal, hypokinesis, and akinesis. There were 14 akinesis segments, 320 normal segments, and 227 hypokinesis segments among the patients.

**Table 2:** Distribution of regional wall motion scores by segment determined using 2DE (*n* = 33)

2DE regional wall motion score group	No. of segments																	
	Basal						Mid						Apical					Total
	AS	A	AL	IL	I	IS	AS	A	AL	IL	I	IS	A	L	I	S	Apical cap	
Normal	22	24	27	24	22	20	16	16	20	22	18	17	13	17	16	13	13	320
Hypokinesis	9	8	6	7	10	12	16	17	13	10	15	16	19	15	17	18	19	227
Akinesis	2	1	0	2	1	1	1	0	0	1	0	0	1	1	0	2	1	14
Dyskinesis	0	0	0	0	0	0	0	0	0	0	0	0	0	0	0	0	0	0

*2DE* two-dimensional echocardiography, *AS* anteroseptal, *A* anterior, *AL* anterolateral, *IL* inferolateral, *I* inferior, *IS* inferoseptal, *L* lateral, *S* septal

#### Use of 3DE SVEF to analyze differences among normal, hypokinesis, and akinesis segments

**Fig. 3:**
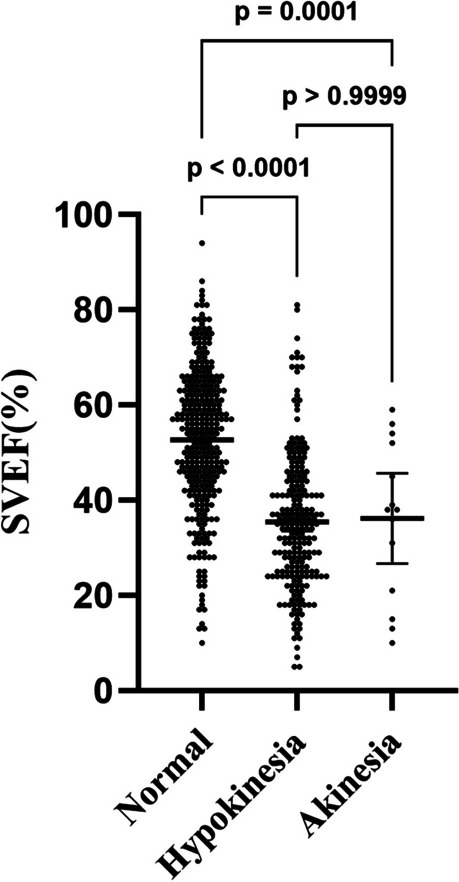
Comparison of three-dimensional echocardiography segmental volume ejection fraction (SVEF) for two-dimensional echocardiography regional wall motion scores using one-way analysis of variance with post hoc analysis and Bonferroni correction

ANOVA was performed to determine whether 3DE SVEF values can be used to differentiate among normal, hypokinesis, and akinesis segments. The analysis showed a significant difference between normal 3DE SVEF values and hypokinesis and akinesis values (*F* = 93.84; *P* < 0.001) but not those of between hypokinesis and akinesis (Fig. 3). After reclassifying hypokinesis and akinesis segments as abnormal, 3DE SVEF values for abnormal and normal segments were significantly different (Table 3).

**Table 3:** Comparison of 3DE SVEF with 2DE for normal or abnormal segments

	2DE result		*P*-value
	Normal segment	Abnormal segment	
3DE SVEF (%)	52.7 ± 15.0	35.5 ± 14.4	< 0.001

Values are presented as mean ± standard deviation. Hypokinesia and akinesia segments were classified as abnormal

*3DE* three-dimensional echocardiography, *SVEF* segmental volume ejection fraction, *2DE* two-dimensional echocardiography

#### Cutoff value for abnormal 3DE SVEF

**Fig. 4:**
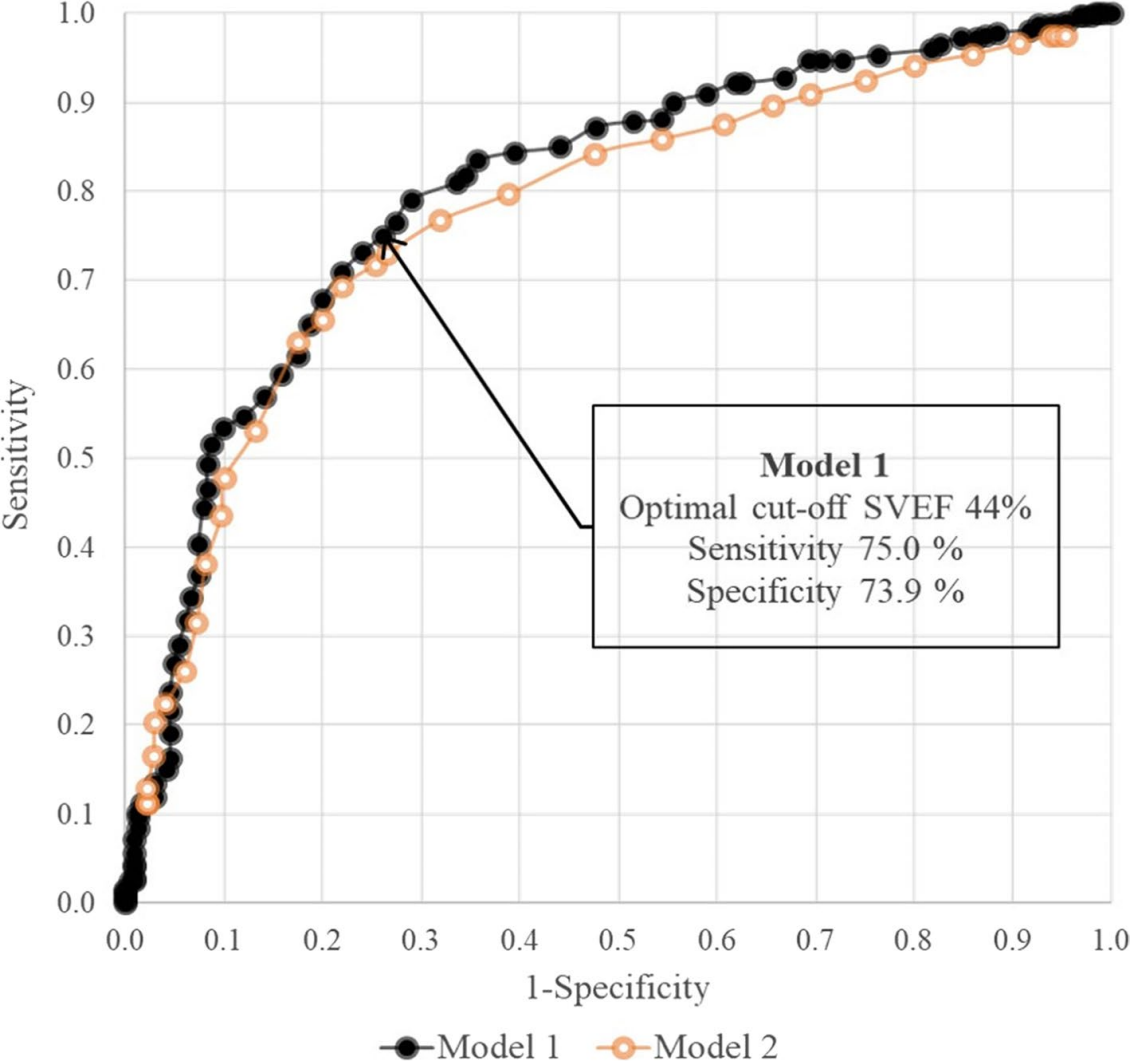
Receiver operating characteristic (ROC) curves of models 1 and 2

ROC curves for models 1 and 2 are provided in Fig. 4. Because model 1 had a larger AUC than the model 2, model 1 was considered to have a better diagnostic performance. Based on the model 1 ROC curve, the optimum SVEF cutoff value was 44%, with a sensitivity of 75.0% and a specificity of 73.9% (AUC, 0.801; 95% CI, 0.763 to 0.838; *P* < 0.001). The model 2 AUC was 0.775 (95% CI, − 0.201 to 1.750; *P* = 0.581).

#### Reliability of predicting coronary territory ischemia using 3DE bull’s-eye polar maps

The results of coronary angiography were 100% consistent with the 2DE predictions. A physician blind to 2DE bull’s-eye polar maps examined 3DE SVEF-derived bull’s-eye polar maps of the study participants and identified culprit coronary territory based on typical coronary artery distribution (Fig. 1). The reliability of 3DE-derived bull’s-eye culprit coronary artery prediction was 81.8% (κ = 0.672; 95% CI, 0.555 to 0.789; *P* < 0.001) (Table 4).

**Table 4:** Reliability results of 3DE-derived bull's-eye culprit coronary artery prediction compared with 2DE

Results of 3DE-derived bull's-eye culprit coronary artery prediction	Culprit coronary artery estimated using 2DE			
	LAD	LCX	RCA	TOTAL
LAD	18	1	1	20
LCX	0	4	2	6
RCA	2	0	5	7
Total	20	5	8	33
κ statistic	0.672			

*3DE* three-dimensional echocardiography, *2DE* two-dimensional echocardiography, *LAD* left anterior descending artery, *LCX* left circumflex coronary artery, *RCA* right coronary artery

#### Interobserver variabilities of 3D endocardial shells and SVEF analysis

Two examiners independently generated 3D endocardial shells and 3DE SVEF values and calculated ICCs for the SVEFs for each segment. Significant interobserver variability was not observed (ICC = 0.714; 95% CI, 0.655 to 0.762; P < 0.001).

#### Interobserver and intraobserver variabilities of 3DE-derived bull’s-eye predictions of culprit coronary arteries

Interobserver and intraobserver variabilities of 3DE-derived bull’s-eye culprit coronary arteries were 97.0% (κ = 0.947; 95% CI, 0.894 to 0.100; *P* < 0.001) and 93.9% (κ = 0.897; 95% CI, 0.827 to 0.967; *P* < 0.001), respectively.

## Discussion

This study showed that 3DE segmental volume analysis could quantify regional LV function. Furthermore, 3DE SVEF values were significantly lower for segments showing RWMA on 2DE. After choosing a cutoff point of 44% for an abnormal 3DE SVEF, determined based on ROC curve analysis, 3DE-derived bull's-eye map coronary artery estimations showed significant agreement with 2DE estimations, which are the clinical gold standard for diagnosing IHD.

Visually determined 2DE RWMA is highly sensitive and used as a reference test [7] for imaging diagnosis of IHD and was a reference in this study. The abilities of other imaging modalities such as Doppler tissue imaging [8] and speckle tracking echocardiography [9] to predict the presence of coronary artery disease and overcome disadvantages of 2DE visual assessments have been evaluated. However, these are rarely used in clinical practice due to their limitations including lack of reference values, poor reproducibility, and significant intervendor variability in measurement [1, 10–12]. In contrast, the results of the present study were consistent with previous studies indicating that 3DE segmental volume analysis could potentially be used for quantitative analysis of RWMA. In addition, semiautomated segmental volume analysis showed the advantages of high objectivity, low interobserver variability, and excellent reproducibility. Segmental volume analysis may accurately reflect segmental RWMA because these volumes reflect the multidirectional length changes in the myocardium.

The analysis showed a significant difference between normal 3DE SVEF values and those for hypokinesis and akinesis. However, as shown in Fig. 3, the difference between hypokinesia and akinesia was nonsignificant in this study, as in a previous study [4]. Because only 14 akinesis segments were included in this study, statistical significance may not have been demonstrated.

**Fig. 5:**
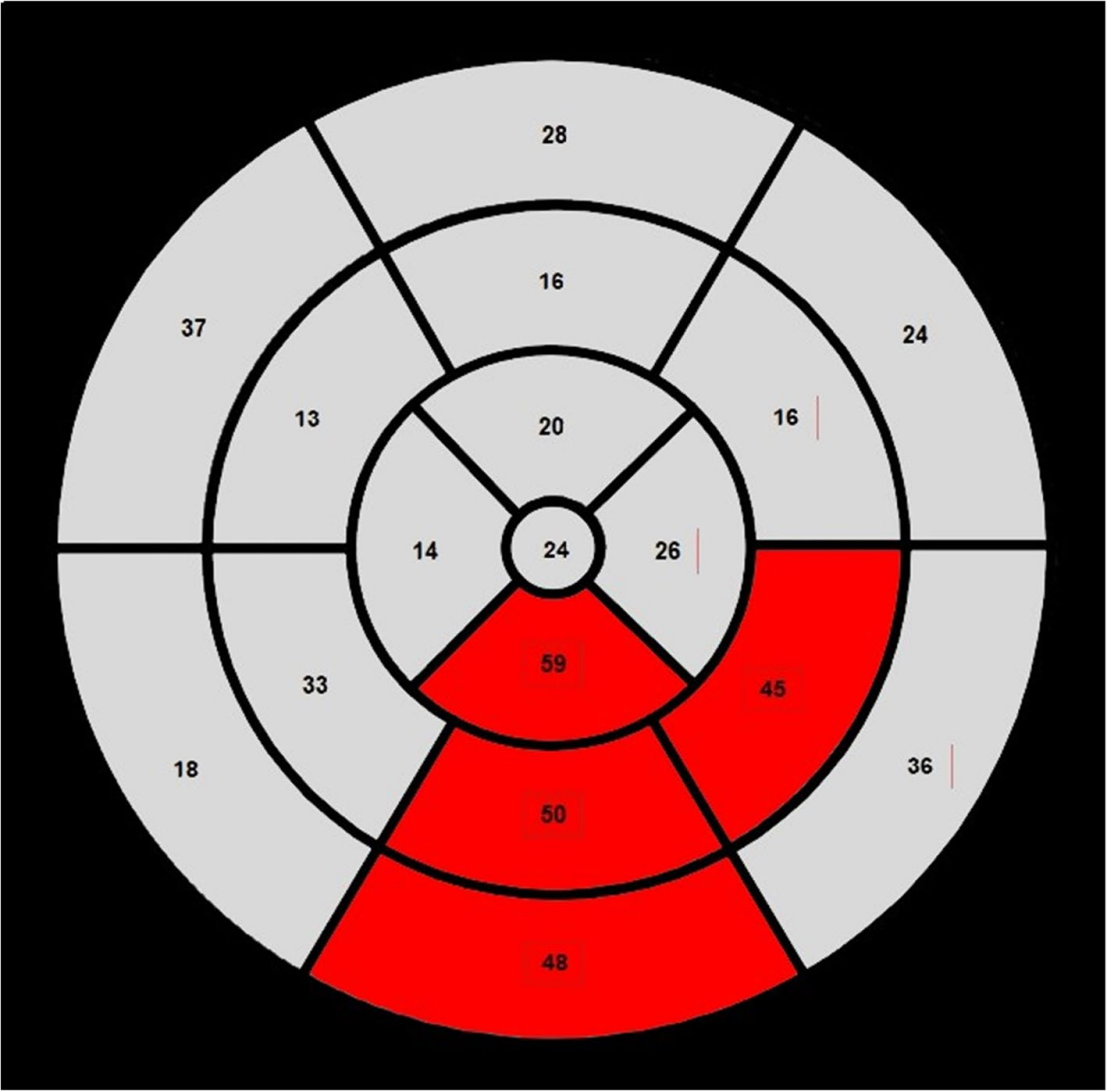
A typical case of three-dimensional segmental volume ejection fraction and bull's-eye model for a patient with left ventricular ejection fraction 20%. Segmental volume ejection fractions of normal segments do not fall within the abnormal range. The segments that could show RWMA were marked in grey, while those without RWMA were marked in red. RWMA, regional wall motion abnormality

Because a standardized SVEF cutoff value has not been determined, we set the cutoff value of abnormal 3DE SVEF at 44% using ROC analysis. There was a concern that applying a fixed SVEF cutoff value might be inappropriate for patients with reduced global EF. For example, if a 44% SVEF cutoff point was determined for a patient with a global LVEF of 30%, the entire SVEF could be considered abnormal. Therefore, we explored the use of an individualized cutoff approach, expressed as a percentage of global EF, to identify the optimal value for assessing RWMA. However, it is incorrect to assume that all SVEFs are lower than the global LVEF (Fig. 5). We analyzed our data to determine the concordance rate of an SVEF cutoff value of 44% in cases where the global EF is either below or above 40%. For the group with a global EF < 40% (*n* = 12), the concordance rate was 91.6% (κ = 0.636; 95% CI, 0.009 to 1.264; *P* = 0.021). For subjects with a global EF ≥ 40% (*n* = 21), the concordance rate was 76.1% (κ = 0.628; 95% CI, 0.349 to 0.907; *P* < 0.001). These results suggest substantial prediction accuracy in both groups.Additionally, we analyzed our data to determine how the SVEF cutoff values vary in cases where the global EF is either below or above 40%.When the absolute fixed cutoff for two subgroups of global EF greater and lesser than 40% was calculated, the following results were obtained: for the group with EF < 40%, the optimal cutoff was 36% (sensitivity, 0.74; specificity 0.75); for the group with EF ≥ 40%, the optimal cutoff was 46% (sensitivity, 0.70; specificity, 0.80). Further investigation is required in low global EF populations.

**Fig. 6:**
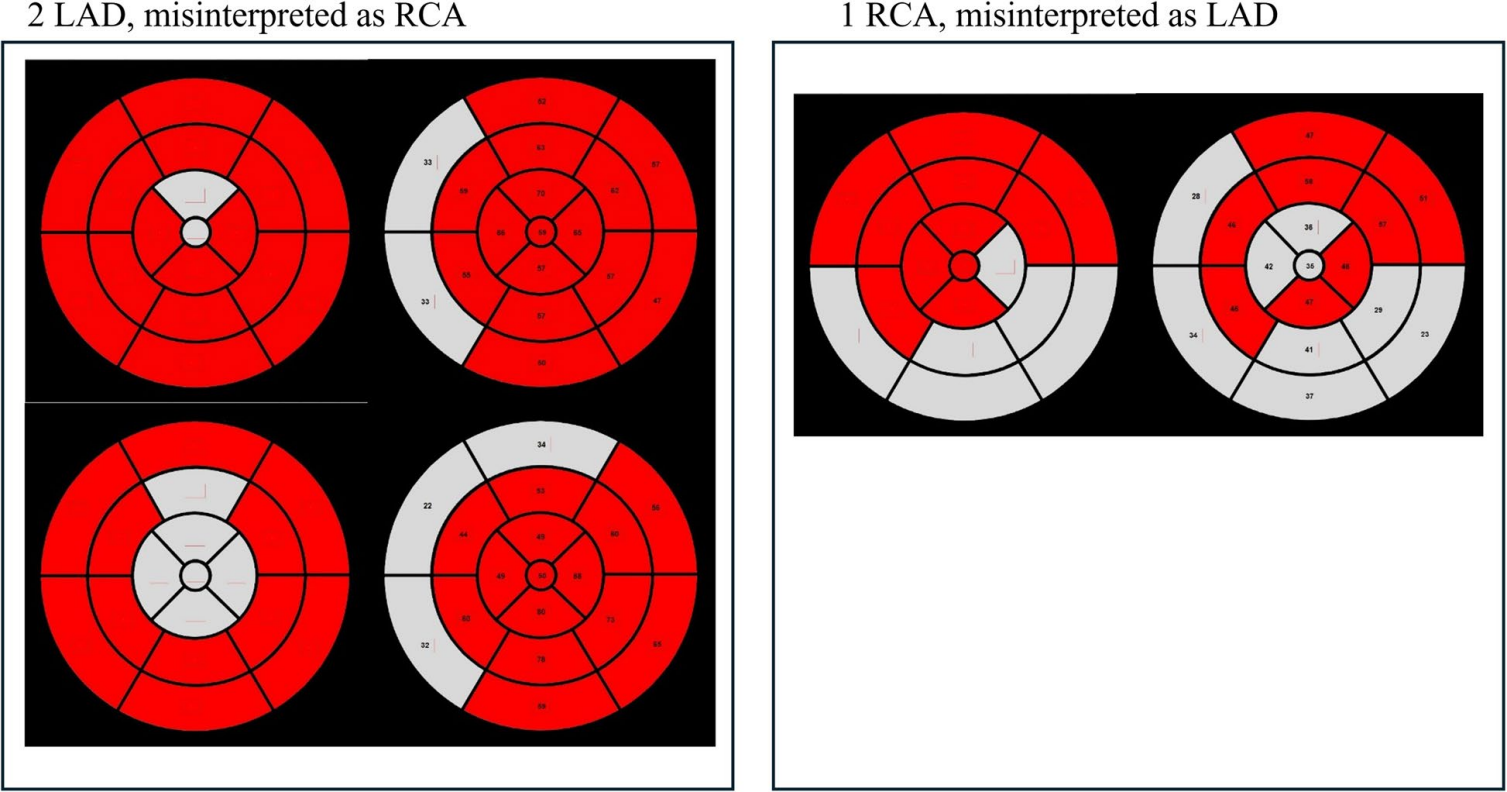
Misinterpreted cases. **A** Two patients with left anterior descending artery (LAD) were categorized as right coronary artery (RCA). **B** One patient with RCA was categorized as LAD

The reliability of 3DE-derived culprit coronary artery prediction was 81.8% (κ = 0.672; 95% CI, 0.555 to 0.789; *P* < 0.001). In the present study, the estimated culprit artery in 2D and in coronary angiography was consistent. Comparing 2D and 3D in this manner can ultimately lead to the same conclusion as when comparing to coronary angiography. In the present study, two patients with left anterior descending artery (LAD) were categorized as right coronary artery (RCA) and one patient with RCA was categorized as LAD (Fig. 6). In general, LAD and RCA abnormalities are significantly different in 2DE. Disagreement was observed in the apex and septal wall segments. In the apical segment, semiautomatic endocardial recognition was difficult when trabeculation was prominent, which was considered the main cause of recognition failure. This is consistent with previous research published by Mor-Avi et al. [6]. Due to the limitation of poor spatial resolution with real-time 3DE, endocardial surface details could not be clearly visualized. This affected the identification of LV boundaries, resulting in significant differences in global and regional volumes, especially the apex. In these cases, technical improvements and further research are needed to solve this problem.

The interobserver discrepancies in 3D endocardial shell generation and SVEF analysis were mainly due to differences in distinction of endocardium and trabeculae due to poor resolution. To improve interobserver variability, technological advancements are needed.

### Limitations

The present study had several limitations. First, there was a high exclusion rate (16%) of subjects due to inadequate imaging, which was caused by a low 3D image frame rate or inadequate spatial resolution, particularly unclear apical wall borders.To resolve this issue, we are considering further research to analyze the SVEF of the LV by rotating images from the subcostal view to avoid the ribs, obtaining focused LV images, and creating clear boundaries of the endocardial wall using contrast 3D transthoracic echocardiography. Second, even considering the pilot nature of this study, the sample size was relatively small, and the study had a retrospective design. Third, 3DE SVEF does not account for myocardial thickening. Fourth, patients with multivessel disease were excluded, and follow-up studies are needed to determine whether the results can be adequately applied to patients with multivessel IHD.

## Conclusions

The results of the present study showed that 3DE segmental volume analysis was feasible to quantify regional LV function, and the prediction of culprit coronary arteries was compatible with those of 2DE and coronary angiography. Therefore, 3DE segmental volume analysis can be used as a quantitative and objective tool for assessing RWMA in patients with IHD.

## Data Availability

No datasets were generated or analysed during the current study.

## Abbreviations

2DE: Two-dimensional echocardiography
3DE: Three-dimensional echocardiography
ANOVA: Analysis of variance
ASE: American Society of Echocardiography
AUC: Area under the curve
EACVI: European Association of Cardiovascular Imaging
EDV: End-diastolic volume
EF: Ejection fraction
ESV: End-systolic volume
ICC: Intraclass correlation coefficient
IHD: Ischemic heart disease
LAD: Left anterior descending artery
LV: Left ventricle
LVEF: Left ventricular ejection fraction
RCA: Right coronary artery
ROC: Receiver operating curve
RWMA: Regional wall motion abnormality
SVEF: Segmental volume ejection fraction
